# 3D-Organotypic Cultures to Unravel Molecular and Cellular Abnormalities in Atopic Dermatitis and Ichthyosis Vulgaris

**DOI:** 10.3390/cells8050489

**Published:** 2019-05-22

**Authors:** Géraldine Leman, Verena Moosbrugger-Martinz, Stefan Blunder, Petra Pavel, Sandrine Dubrac

**Affiliations:** Epidermal Biology Laboratory, Department of Dermatology, Venereology and Allergology, Medical University of Innsbruck, 6020 Innsbruck, Austria; geraldine.leman@i-med.ac.at (G.L.); Verena.Martinz@i-med.ac.at (V.M.-M.); stefan.blunder@i-med.ac.at (S.B.); petra.pavel@i-med.ac.at (P.P.)

**Keywords:** atopic dermatitis, ichthyosis, organotypic culture, epidermal equivalent, skin equivalent, filaggrin

## Abstract

Atopic dermatitis (AD) is characterized by dry and itchy skin evolving into disseminated skin lesions. AD is believed to result from a primary acquired or a genetically-induced epidermal barrier defect leading to immune hyper-responsiveness. Filaggrin (FLG) is a protein found in the cornified envelope of fully differentiated keratinocytes, referred to as corneocytes. Although *FLG* null mutations are strongly associated with AD, they are not sufficient to induce the disease. Moreover, most patients with ichthyosis vulgaris (IV), a monogenetic skin disease characterized by *FLG* homozygous, heterozygous, or compound heterozygous null mutations, display non-inflamed dry and scaly skin. Thus, all causes of epidermal barrier impairment in AD have not yet been identified, including those leading to the Th2-predominant inflammation observed in AD. Three dimensional organotypic cultures have emerged as valuable tools in skin research, replacing animal experimentation in many cases and precluding the need for repeated patient biopsies. Here, we review the results on IV and AD obtained with epidermal or skin equivalents and consider these findings in the context of human in vivo data. Further research utilizing complex models including immune cells and cutaneous innervation will enable finer dissection of the pathogenesis of AD and deepen our knowledge of epidermal biology.

## 1. Introduction

Atopic dermatitis (AD) is one of the most common inflammatory skin diseases worldwide. It is a chronic, relapsing, and pruritic skin disorder [1] which results from complex interactions between epidermal barrier abnormalities and immune dysregulation, aggravated by environmental factors, including stress, food, pollution, pollen, animal dander, and skin microbiota. AD is associated with severe comorbidities such as asthma and allergic rhinitis (atopic march) and imposes on patients a heavy socio-psychological burden leading to social isolation, depression, and suicide ideation in the more severe cases [2]. Early disease onset with full remission before the age of 8 years is observed in most patients, although late onset (>18 years old) and disease persistence into adulthood are both possible. AD is characterized by a generalized dry skin accompanied by acute papulo-vesicular eruption and chronic pruritic eczema. In AD patients, during disease flares, nonlesional and lesional eczematous skin coexist. However, the mechanism of transition from the non-affected to the affected state remains unclear. Thus, AD is believed to result from acquired or genetically induced epidermal barrier weakening with superimposed local aggravating factors leading to skin inflammation, but these factors remain to be fully identified.

AD patients comprise a heterogeneous population with spatial, temporal, morphological, genetic, and immunological differences. However, current knowledge favors a common etiology in which primary epidermal barrier impairment drives skin inflammation and food allergy in AD [3]. The causes of acquired- or genetically induced epidermal barrier impairment in AD are not fully elucidated. Genetic studies have identified AD risk loci in the epidermal differentiation complex, especially in the Filaggrin (*FLG*) gene [4] and in genes within cytokine clusters [5]. AD patients who carry *FLG* null mutations are more prone to develop severe disease symptoms persisting into adulthood [6,7,8]. Phenotypic divergences exist between AD children and AD adults, as do differences in the epidermal levels of expression of antimicrobial peptides (AMPs) and *FLG* [9]. Immune abnormalities in AD are complex and depend on the disease state. However, both extrinsic (presence of allergies) and intrinsic (absence of allergies) AD [10] are considered to be Th2-driven inflammatory skin diseases. Nonlesional AD skin and AD lesions display overlapping features such as epidermal hyperplasia and predominant Th2/Th17 inflammation [11]. Increased production of alarmins (TSLP, IL-33, IL-25, HMGB1, IL-1α) is observed in acute AD skin lesions, whereas chronic AD skin lesions exhibit microbial superinfection associated with abnormal innate and adaptive immunity [11,12]. The skin microbiota is altered in AD, beginning with dysbiosis, in nonlesional AD and culminating in *Staphylococcus*-mediated superinfection in severe forms of AD or upon disease flares [13,14]. A predominance of *Staphylococcus epidermidis* is observed in patients with mild disease, whereas a predominance of *Staphylococcus aureus* is observed in patients with severe disease [15]. Moreover, skin superinfection with viruses (e.g., eczema herpeticum) or fungi (e.g., *Malassezia sympodialis*) is also observed.

Ichthyosis vulgaris (IV) is the most frequent form of ichthyosis and is characterized by heterozygous, homozygous, or compound heterozygous *FLG* null mutations [16]. Other ichthyoses include, inter alia, recessive X-linked ichthyosis, autosomal recessive congenital ichthyosis (ARCI), and keratinopathic ichthyosis. Ichthyoses are characterized by major and, usually, generalized skin scaling. They can be monogenic diseases, but acquired ichthyosis without a known genetic origin can also be observed in patients with cancer, autoimmune, inflammatory, metabolic, endocrine, and infectious diseases, nutritional deficiency, and medication use [17]. Patients with IV display dry and scaly skin but no signs of overt inflammation. The main histological features of IV skin are hyperkeratosis and marked reduction of keratohyalin granules, which can be totally absent in more severe cases [18]. At the ultrastructural level, lower corneodesmosome density as well as impaired loading and secretion of lamellar bodies (LBs), leading to abnormal lamellar bilayer architecture, are observed in IV [18,19]. FLG monomers are believed to mediate the collapse of the keratin (KRT) filament network during cornification. However, data on KRT intermediate filaments are contradictory, with studies showing either normal filaments [20,21] or their perinuclear retraction in IV [19]. Moreover, the amounts of KRT 5, 6 and 1 are not altered in IV keratinocytes (KCs) when compared to healthy cells [21]. IV and AD share several common clinical features such as dry skin and susceptibility to skin superinfection [22,23] as well as cellular abnormalities such as epidermal hyperplasia, weakened tight junctions, and abnormal LBs [19]. However, our recent transcriptomic analysis revealed distinguishable gene expression signatures in AD and IV skin [24].

The past 20 years of research on AD established that it results from a combination of genetic risk factors and immune hyper-responsiveness, both strongly influenced by environmental factors. Although extensive data have been generated utilizing genetics, Omics, and various other biotechnologies, AD pathogenesis has not been sufficiently addressed, leaving many questions unanswered. Difficulty in obtaining tissue from patients, especially young patients, and the limited scope of experiments that can be carried out with biopsy material, complicate the acquisition of clinically relevant data. Moreover, extrapolation of data obtained from mouse models to humans should be made with caution [25]. Regulations limiting the use of laboratory animals on ethical grounds are being implemented worldwide according to so-called 3R principles (Replace, Reduce, and Refine) (Directives of the European Parliament, [26]). Thus, 3D cultures generated with human cells are valuable alternatives to mouse experiments. Although 3D cultures have only recently been applied to study skin diseases, they have already revealed several new pathogenic pathways involved in AD as well as cellular and molecular abnormalities in ichthyoses, including IV. In the near future, generation of more complex 3D skin models will help decipher further relevant pathomechanisms and uncover new therapeutic approaches. Several teams have worked in this direction by creating a panel of models of human epidermis (HEEs) and full-skin (HSEs) equivalents composed of various skin cell types such as Langerhans cells, melanocytes, T cells, macrophages, and endothelial cells [27,28,29,30,31,32]. The differences between HSEs and native skin, including lipid composition, have been recently reviewed by Niehues et al. [33]. Immortalized KCs, such as HaCaT and N/TERT cells, can also be advantageously utilized for drug testing because of improved standardization owing to their unlimited availability and avoidance of genetic and morphological inter-individual variability [34,35]. Moreover, human mesenchymal stem cells from adipose tissue or bone marrow [36] or germline-derived pluripotent stem cells [37] have been employed to generate or improve HSEs. Recently, HEEs have been established with induced pluripotent stem cells (iPSCs) and compared with HEEs generated from healthy primary KCs, revealing no major differences in morphology, expression of differentiation markers and epidermal barrier function [38,39]. To avoid having to obtain skin punch biopsies, hair follicle-derived KCs can be prepared, possibly immortalized and utilized in HEEs [40,41]. Recently, Muller et al. engineered complex 3D organotypic cultures in which iPSCs, generated from human skin fibroblasts, were re-differentiated into sensory neurons and Schwann cells [42]. This innovative innervated HSE, when used with patient-derived cells, could potentially help in investigating the role of cutaneous innervation in a broad range of skin diseases [42].

## 2. 3D Cultures to Study Cellular and Molecular Abnormalities in Ichthyosis Vulgaris and Beyond

Several models have been employed to study IV, including HEEs and HSEs generated with immortalized cells stably or transiently knocked down for *FLG* or with patient or control primary cells.

IV is characterized by dry and scaly skin mainly of the lower abdomen, arms, and legs, palmar hyperlinearity, and keratosis pilaris. Absent or reduced keratohyalin granules in the epidermis and hyperkeratosis are the main histological features found in IV. IV is a monogenetic disease originating from *FLG* null mutations. *FLG* is located in the epidermal differentiation complex on chromosome 1q21 [43]. Loss-of-function mutations in *FLG* have been identified in many populations around the world. Their frequency and type (e.g., p.R501X and c.2282del4 in northern Europe, p.S2554X and c.3321delA in Japan) vary among populations [16]. FLG is a 37 kDa cytoplasmic protein produced after dephosphorylation and proteolytic cleavage of its precursor, namely proFLG (~400 kDa) localized in keratohyalin granules, into 10 to 12 monomers [44]. FLG is believed to aggregate and align KRT bundles, hence contributing to the integrity and mechanical resilience of the stratum corneum (SC) [43]. In the SC, FLG is processed by various proteases into *trans*-urocanic acid (UCA) and pyrrolidone carboxylic acid (PCA), which are components of natural moisturizing factors (NMFs) together with lactate, chloride and sodium ions, and urea. NMFs contribute to skin hydration and establishment of a surface acidic pH, thereby limiting the overgrowth of microbes [45].

### 2.1. Absence of Keratohyalin Granules and Subtle Alterations in Lipid Composition in FLG-Deficient 3D Cultures

**Figure 1:**
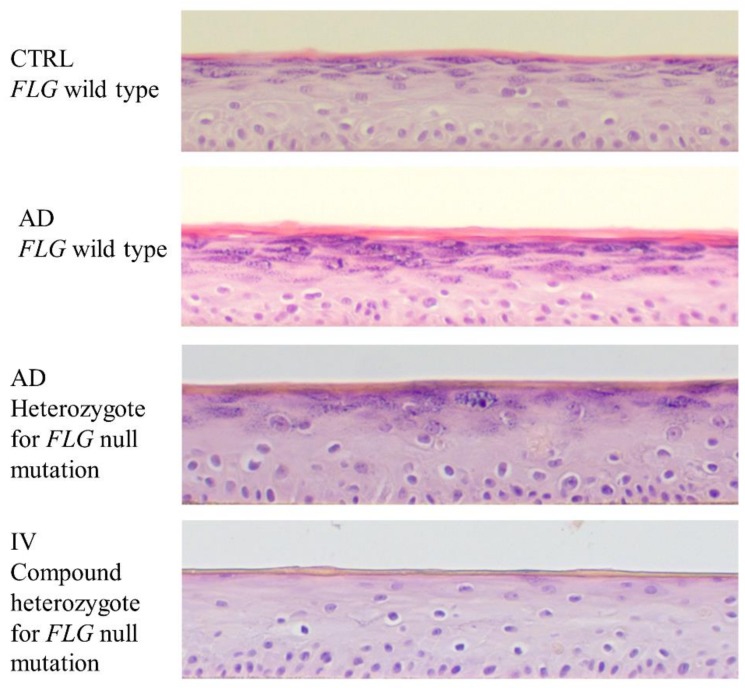
Morphology of atopic dermatitis (AD) and ichthyosis vulgaris (IV) human epidermal equivalents (HEEs). Representative images of HEEs generated with keratinocytes from healthy donors (CTRL) and from patients with AD and/or IV stained with hematoxylin and eosin.

Mildner et al., by using HSEs and siRNA for *FLG*, observed loss of keratohyalin granules in the stratum granulosum (SG) and abnormal LB formation [46], similar to findings reported in HEEs where *FLG* was knocked down by shRNA [47] or generated with IV patient primary KCs [48,49], and in the epidermis of IV patients [19]. No alterations in the total amounts of skin lipids, including cholesterol, cholesterol esters, ceramides, triglycerides, glucosylceramides, sphingomyelin, and phospholipids, have been observed [46,50,51]. However, Wallmeyer et al. found increased amounts of glucosylceramides and a decreased ratio of omega-acylceramide CER[EOS]/CER[EOdS] in HSEs knocked down for *FLG* [51]. This was associated with reduced SC lipid organization, as measured by infrared spectroscopy [50,51]. Either no alterations in epidermal free fatty acid (FFA) content [46,52] or increased amounts of epidermal FFAs associated with increased sPLA_2_ [50,51] were observed in HSEs knocked down for *FLG*. We have shown that HEEs generated with KCs isolated from IV patients (Figure 1) recapitulated the abnormalities observed in the epidermis of IV patients, i.e., disorganized lamellar bilayer structures. This latter phenotype results from disruption of the mature lamellar bilayer organization, owing to incompletely processed lamellar material, aberrant LB internal structures and LB entombment, premature secretion of LB contents into the extracellular spaces of the SG, and inhomogeneous secretion at the SG-SC interface [19,49]. Overall, these studies show that subtle changes in epidermal lipid composition observed in FLG deficiency are sufficient to disorganize SC lipids and alter LB formation, albeit other factors such as the F-actin cytoskeleton might also be involved [53].

### 2.2. The Efficacy of FLG Knockdown in 3D Cultures Correlates with SC Barrier Fitness

FLG deficiency is believed to provoke epidermal barrier impairment and, in turn, enhanced permeability to chemicals and allergens. For ethical reasons, verifying this hypothesis in vivo is impossible. Thus, FLG-deficient HEEs or HSEs are valuable tools to evaluate the ability of FLG deficiency to increase the permeability of the SC (outside-in barrier) to various substances and extrapolate potential leakiness of the SC to IV patients. When Küchler at al. applied sodium dodecyl sulfate, an anionic surfactant used in many cleaning and hygiene products and able to disrupt the SC barrier at high concentrations, onto HSEs efficiently knocked down for *FLG*, they detected elevated release of lactate dehydrogenase and pro-inflammatory mediators, i.e., IL-6 and IL-8, into the culture medium [54]. This suggests that FLG deficiency increases both the fragility and permeability of the SC. However, more direct measurements of SC permeability can be performed by using various molecules, of which the physicochemical nature (hydrophilic vs. hydrophobic) is crucial. Lipophilic compounds penetrate HSEs or HEEs regardless of the quality of the SC barrier, and only the quantity and the lag time are impacted, notably by the number of SC layers and the nature of the structural SC ceramides [55]. In contrast, hydrophilic compounds penetrate into the epidermis only upon SC damage [56,57,58], so that the more compound that penetrates into the culture, the more severe the epidermal barrier impairment [57]. Thus, one can conclude that if FLG deficiency alters the number of SC layers and/or the nature of the SC ceramides, then penetration of hydrophilic compounds should not be affected, i.e., they will not cross the SC barrier and penetrate into the living layers of the epidermis; conversely, penetration by lipophilic compounds should be enhanced and accelerated. In contrast, if FLG deficiency inflicts major damage to the SC barrier, then hydrophilic compounds should penetrate into the epidermis and the penetration by lipophilic compounds should be drastically accelerated. Penderies et al. observed major SC impairment in HEEs knocked down for *FLG*, as evidenced by the diffusion of Lucifer Yellow (LY) (hydrophilic) as far as the basal layer [47]. This likely resulted from thinning of the epidermis and of the SC, absence of corneodesmosomes, and an altered corneocyte intracellular matrix [47]. In contrast, others showed that percutaneous absorption of lipophilic drugs was higher than for hydrophilic drugs [50,54], suggesting attenuated effects of *FLG* knockdown on the structure of the SC. van Drongelen et al. generated HEEs with immortalized (N/TERT) KCs knocked down for *FLG* and observed normal epidermal and SC thickness and SC lamellar lipid organization, consistent with a lack of permeability to butyl para-aminobenzoic acid (butyl-PABA), a lipophilic compound, and thereby demonstrating no alteration of the SC barrier in their model [52]. Similar results were obtained with HSEs knocked down for *FLG* and exposed to testosterone (lipophilic) [59]. Thus, *FLG* knockdown in HSEs and HEEs has produced a complete spectrum of results, ranging from major SC barrier damage to no noticeable alteration. Work with HEEs generated with IV patient primary KCs showed no alteration of percutaneous absorption of hydrophilic (LY) or lipophilic (testosterone) compounds [48,49]. In patients with IV, a reduction but not a total disappearance of corneodesmosomes associated with abnormalities of lamellar bilayer architecture and a thickened epidermis and SC have been observed [19]. Thus, in IV patients, hyperkeratosis might largely impede the penetration of molecules into the living layers of the epidermis, despite abnormalities in the SC. Indeed, increased penetration by allergens and xenobiotics into the skin of IV patients has not yet been demonstrated. The crescendo of effects observed in 3D cultures knocked down for *FLG* might simply reflect the quantity of residual FLG and the higher vulnerability of such cultures to *FLG* knockdown when compared to patient skin, with Pendaries et al. and Wang et al. achieving the most effective *FLG* knockdown (90% at the mRNA and protein levels) [47,50,52,60]. These observations raise questions about the suitability of such ultra-efficient *FLG* knockdown experiments to study the role of FLG deficiency in ex vivo models, which might be more fragile than skin and lack compensatory mechanisms that may exist in vivo. In this case, the better might be the enemy of the good. Another important technical point to underline when studying epidermal barrier function using HEEs concerns the culture conditions and especially the humidity. In newborn babies, skin exposure to lower ambient humidity enables the maturation of the epidermal barrier [61]. Similarly, the quality of the epidermal barrier of HEEs also depends on ambient humidity, which can influence several parameters important to barrier establishment. Recently, Sun et al. demonstrated major morphological differences in HEE cultures at 50% humidity when compared to those cultured at 90% humidity, including more SC layers and a higher density of corneodesmosomes [62]. However, the expression of most differentiation and tight junction markers as well as genes related to lipid metabolism and AMPs was similar in both culture conditions, with the exception of *FLG* and two osmolyte regulators [62]. Similarly, culture temperature (33 °C versus 37 °C) can moderately affect the expression of epidermal differentiation markers and the composition of SC ceramides [63]. Thus, in the future, both the humidity and temperature of 3D cultures should be more carefully considered, especially for studying epidermal barrier function.

### 2.3. Higher Porousness of FLG-Deficient 3D Cultures to Staphylococcus aureus

The quality of the epidermal barrier shapes the skin microbiota, and defective barrier function can lead to expansion of microbes on the skin. With the current fervor about the skin microbiome and its potential implication in AD, several groups have begun developing 3D models inoculated with *Staphylococcus aureus*. Indeed, considerable emphasis has been brought to this bacterium, which is suspected of contributing to AD pathogenesis and flares [13,14,64,65,66]. By using HSEs generated with healthy KCs, Nakatsuji et al. showed that *Staphylococcus aureus* can penetrate into the epidermis and reach the dermis 48 h after inoculation [67]. Moreover, *FLG* knockdown in HEEs favors adherence of *Staphylococcus aureus* to the SC, which results in the release of more IL-8 [68], despite increases of both human-β-defensin (HBD) 2 and HBD3 at the mRNA and protein levels [59,68]. Upregulation of *HBD2* was not mediated via IL-1R, IL-6, TLR2, or TSLP [59]. This work suggests a higher porousness of FLG-deficient HEEs to *Staphylococcus aureus* and potentially an increased susceptibility of IV patients and of AD patients bearing *FLG* null mutations to this specific bacterium. However, recent work on the skin microbiota of IV patients tends to disfavor this hypothesis [69].

### 2.4. FLG Deficiency Has Contrasting Effects on Tight Junctions and KC Differentiation in 3D Cultures

The epidermal inside-out barrier mainly relies on tight junctions, and its efficacy can be evaluated by measuring transepithelial electrical resistance (TEER) and the diffusion of EZ-Link Sulfo-NHS-LC-LC-Biotin in HSEs/HEEs [70]. Interestingly, it is possible to disrupt tight junctions in HSEs by applying GST-fused C-terminal half of *Clostridium perfringens* enterotoxin (GST-C-CPE), a polypeptide with inhibitory activity against claudin (CLDN)-4 and CLDN-6 [71]. In such HSEs, the outside-in barrier (SC barrier) is also affected, accompanied by immature lamellar structures, a less acidic surface pH, absence of keratohyalin granules and reduced amounts of FLG monomers [72], i.e., a constellation of observations associated with IV. Wang et al. showed nearly a doubling of TEER in HEEs generated with *FLG* knocked down HaCaT cells, demonstrating an impaired inside-out barrier [60], whereas others, using HEEs generated with KCs taken from IV patients, did not find any abnormalities in the inside-out barrier, as measured with diffusion of EZ-Link Sulfo-NHS-LC-LC-Biotin [48]. Moreover, Wallmeyer et al. and Hönzke et al. found increased amounts of occludin (OCLN), a tight junction protein, in HSEs knocked down for *FLG* [51,59]. Results on CLDN-1 were more inconsistent, with data showing increased or unchanged amounts [51,59]. The expression of tight junction proteins is linked to proper KC differentiation. For Mildner et al. and van Drongelen et al., KC differentiation was not altered [46,52], whereas for others, it was triggered based on the increased amounts of involucrin (IVL) and loricrin (LOR) found in HSEs knocked down for *FLG* [51]. In contrast, Pendaries et al., Küchler et al. and Wang et al. found impaired KC differentiation [47,54,60]. Moreover, Wang et al. showed thickening of the epidermis associated with increased KC proliferation and enhancement of endogenous cysteine protease and cathepsin B activities [60]. In that work, zonula occludens (ZO)-1 and CLDN-1 were reduced and this was associated with abnormal KC differentiation, as evidenced by lower amounts of KRT1 and 10. Treatment with inhibitors of cysteine protease restored the efficacy of tight junctions via re-induction of the expression of tight junction proteins [60]. These results are in line with work from Aho et al., where *FLG*-silenced HSEs displayed thicker epidermis, potentially due to an abnormal differentiation process, as evidenced by increased KRT16, and despite normal quantity and distribution of KRT14, KRT10, E-cadherin, β-catenin, and IVL [73]. Our group generated HEEs with IV patient KCs and did not find any gross abnormalities in epidermal differentiation, only a strong decrease in hornerin (HRNR) at both the mRNA and protein levels, suggesting a link between FLG and HRNR [49]. Indeed, HRNR has been detected at the periphery of keratohyalin granules [74]. Both FLG and HRNR are fused S100 proteins encoded by the epidermal differentiation complex and components of the cornified envelop (CE) and cross-linked to KRT filaments. FLG is a substrate of transglutaminase (TGM) 1, the first step of CE formation, whereas HRNR is a substrate of TGM3, the second step of CE formation. Several studies focusing on epidermal barrier dysfunction showed parallel downregulation of FLG and HRNR [75,76,77]. Th2 cytokines (i.e., IL-4, IL-13, and IL-25) have been shown to downregulate FLG and HRNR and thus to be causative for the strong reduction of both proteins in AD skin [77]. Our work has shown that both proteins were strongly reduced in IV HEEs, where no Th2 cytokine was added, suggesting that another mechanism is involved [49]. A possible mechanism would be proteolytic processing via the same enzyme (Calpains I and II) [78,79]. Calpains are activated by various stimuli, including calcium and epidermal growth factor (EGF) [80], whose variations have not yet been investigated in IV. Thus, further work is required to better understand the relationship between FLG and HRNR. In patients with IV, transepidermal water loss (TEWL) is significantly increased but this increase is very subtle and remains in a non-pathological range (i.e., <10 g water/h/m^2^ skin) [19]. Moreover, IV patients exhibit hyperplastic epidermis owing to increased KC proliferation. The expression of differentiation markers other than FLG has not yet been studied in IV patients but is likely to show little or no alteration. In fact, the morphology of the suprabasal epidermal layers and the amounts of various differentiation markers in the SC of IV patients are barely affected, if at all [81]. Thus, FLG deficiency, per se, might have minor effects on tight junctions, although further work is required to better define any relationship. Furthermore, the contrasting effects of *FLG* knockdown in HSEs and HEEs on KC differentiation and, in turn, on tight junctions, together with discrepancies in the cellular abnormalities observed in IV patients, might also relate to the efficacy of *FLG* knockdown.

### 2.5. FLG-Deficient 3D Cultures: Reduced NMFs but Increased 15-HETE

*FLG* knockdown in HSEs or HEEs has consistently led to reduced amounts of UCA and PCA in the SC [46,47,50], but with unaltered surface pH [50] even though these components of NMFs are believed to contribute to the skin’s acidic pH [82]. Indeed, skin pH might be maintained by normal activity of sodium-hydrogen antiporter 1 (NHE-1). NHE-1 is a ubiquitous sodium proton exchanger involved in the maintenance of physiological intracellular pH and the control of acidification of extracellular domains in the SC [83]. Mice deficient for the histidase enzyme responsible for the conversion of FLG into histidine, which is the first step of its conversion to UCA, did not display changes in SC acidity, possibly due to compensatory NHE-1 and sPLA2 upregulation [84]. In contrast, deficiency in either NHE-1 or sPLA2 led to dissipation of SC acidity, indicating their importance and complementarity in establishing SC acidity [83,85,86]. In IV, a small increase in skin pH was observed in patients homozygous or compound heterozygous for *FLG* null mutations [19]. Thus, it is likely that reduced amounts of UCA and PCA, as observed in FLG-deficient skin or in ex vivo models, per se, have little or no effects on the acidity of SC pH. In contrast, UCA, after conversion into *cis*-UCA, has been postulated to act as a “natural” sunscreen [87]. Consistent with this, *FLG* knockdown leads to increased sensitivity of KCs to UVB [46,47]. These results tend to support the beneficial effects of UCA as a natural barrier against sun irradiation.

Generating HEEs with patient KCs helped unravel previously unidentified cellular and molecular abnormalities in IV. Eicosanoids serve as important bioactive signaling molecules in the epidermis [88,89]. We found that 15-hydroxyeicosatetraenoic acid (HETE) and 15*S*-hydroxy-5*Z*,8*Z*,11*Z*,13*E*,17*Z*-eicosapentaenoic acid (HEPE) accumulated in IV HEEs [49]. 15-HETE is produced in KCs upon UVB irradiation where it can inhibit 12-lipoxygenase (LOX) activity and thus the production of pro-inflammatory lipid mediators [90,91]. Hence, it was postulated that 15-HETE helps KCs to resist damage inflicted by sunburn [92,93]. This is of potential interest because FLG deficiency leads to reduced levels of UCA and PCA, which protect the skin from sun irradiation. Thus, KCs from IV patients might have adapted to and compensated for this lack of “natural” sunscreen by upregulating the production of 15-HETE. Moreover, this specific lipid inhibits T-cell proliferation and reduces the synthesis of leukotriene B4 by leukocytes [94,95,96,97,98]. Thus, prophylactic or therapeutic stimulation of 15-LOX or application of topical ointments containing 15-HETE might be protective against sunburn, especially in patients with FLG deficiency.

### 2.6. Increased Secretion of TSLP in FLG-Deficient 3D Cultures

IV patients, despite having dry skin, do not exhibit overt inflammation [18]. Accordingly, HEEs generated with KCs isolated from IV patients did not exhibit upregulation of pro-inflammatory mediators (*IL1B*, *TNFA*, *TSLP*, *CCL17*, *IL33*), at least at the mRNA level [49]. However, large numbers of CD4^+^ T cells migrated into the dermis when added underneath *FLG* knocked down HSEs, potentially due to increased TSLP production, which was not observed in control HSEs [51]. This is consistent with another study showing increased TSLP in *FLG* knockdown HSEs [59]. TSLP is an alarmin produced by damaged KCs. Thus, it is possible to speculate that *FLG* knockdown might more profoundly affect KCs than do inherited loss-of-function mutations, potentially via adaptive or compensatory mechanisms that do not have time to establish in healthy KCs silenced for *FLG*.

### 2.7. ARCI 3D Cultures to Test Enzyme Replacement Therapy

Autosomal recessive congenital ichthyosis (ARCI) is a heterogeneous group of rare disorders of cornification, including lamellar ichthyosis, congenital ichthyosiform erythroderma, and harlequin ichthyosis. Newborns with ARCI display a collodion membrane and patchy or generalized scaling, evolving into large scaly plaques associated with erythema. However, nearly normal-appearing skin can also be observed in some older patients. Ten genes are currently known to be associated with ARCI: *TGM1*, *ALOXE3*, *ALOX12B*, *NIPAL4*, *ABCA12*, *CYP4F22*, *PNPLA1*, *CERS3*, *SDR9C7*, and *SULT2B1*. Mutations in *TGM1* account for most ARCI cases. TGM1 is a catalytic membrane-bound enzyme involved in terminal epidermal differentiation and thus in formation of the cornified cell envelope and SC barrier. Most TGM1 is anchored to the KC plasma membrane, where it covalently crosslinks omega-hydroxyceramides by ester bonds to CE proteins, mainly IVL but also FLG. In line, ultrastructural analysis of the SC from patients with TGM1 deficiency demonstrates missing or attenuated CEs, but normal cornified lipid envelope, corneodesmomes, and LBs. TGM1 deficiency-induced ichthyosis is characterized by impairment of epidermal barrier function, resulting in frequent infections. Eckl et al. developed HSEs with cells isolated from ARCI patients or with KCs silenced for *TGM1* that recapitulated the main features of the disease, including massive thickening of the SC [99]. They utilized the same strategy (i.e., siRNA) to silence *ALOX12B*, *ALOXE3*, *NIPAL4*, *CYP4F22*, or *ABCA12* in HSEs [99]. In HSEs with knocked down *ALOX12B*, *ALOXE3,* or *NIPAL4*, they observed reduced amounts of FLG as well as increased permeability to LY [99], suggesting major impairment of the SC barrier. Recently, in a follow-up study, the same group utilized *TGM1* knocked down HSEs to test the effectiveness of TGM1 replacement therapy [100]. This is of great importance because no cure exists for patients with monogenetic ichthyoses. Because the phenotype of all these patients, regardless of age, can be quite severe, provoking not only pain but also social isolation, it is mandatory to find efficacious therapeutic options. Moreover, such studies can serve as models for other monogenetic diseases as well as lay the groundwork for novel therapeutic approaches for more complex diseases. In their work, Plank et al. topically applied TGM1 loaded in a thermoresponsive nanogel (dPG-pNIPAM tNGs) which allows effective delivery through the SC [101]. They observed not only TGM1 in the granular layer of *TGM1* knocked down epidermis but also its activity [100]. A similar strategy was successfully deployed by Stout at al. to replenish FLG in FLG-deficient epidermis, with the ultimate goal to cure IV and alleviate AD symptoms [102]. The authors used a recombinant FLG monomer protein covalently linked to the human immunodeficiency virus trans-activator of transcription (Tat)-derived cell-penetrating peptide motif, which was able to penetrate the epidermis, be internalized by KCs and processed to an appropriate size after topical application [102]. Thus, these promising results could pave the way to novel therapies to treat incurable genetic skin diseases such as ichthyoses and beyond.

## 3. 3D Cultures Helped Unravel New Pathogenic Pathways in Atopic Dermatitis

Several strategies have been utilized to study AD in HEEs and HSEs. This has helped unravel new mechanisms involved in AD pathogenesis and test new therapeutic approaches. Some groups have generated 3D organotypic cultures with control KCs treated with a cytokine cocktail, whereas others have knocked down *FLG* and added a cytokine cocktail or employed primary patient cells. Cytokine cocktails usually contain IL-4 and IL-13 because these are two major Th2 cytokines found in the skin of AD patients, regardless of disease stage (nonlesional and acute/chronic lesional) [12]. Treatment of HSEs with IL-4 and IL-13 induced epidermal hyperplasia (acanthosis) and a reduction of FLG, which are two key cellular abnormalities associated with AD [103,104,105]. However, this treatment induced a thinning of the SC as well, which is opposite to the hyperkeratosis observed in AD [103,104]. Moreover, IL-4 alone was able to dampen epidermal differentiation by downregulating FLG, IVL, LOR, *HRNR*, *KRT10*, *KRT14*, and *KLK7* and exerted contrasting effects on markers of inflammation (upregulation of *IL33* and *CCL26*, downregulation of *IL18* and *IL1RL1*) [104]. Although FLG is degraded into acidic compounds in the SC, its absence from the epidermis does not lead to increased skin surface pH (see above). In contrast, addition of IL-4 and IL-13 to HSEs led to alkalinization of the surface pH, regardless of FLG [59]. Moreover, when IL-4 and IL-13 were added to *FLG* knocked down HSEs, the amounts of OCLN and HBD2 were decreased, whereas this effect was not observed in *FLG* wild-type models [59]. Thus, these results suggest that the immune environment found in AD skin synergizes with FLG deficiency to further alter key parameters of the epidermal barrier, i.e., tight junctions and innate immunity.

### 3.1. Alarmins Affect the Proliferation/Differentiation Balance in Keratinocytes but do not Trigger Inflammation in 3D Cultures

Alarmins (TSLP, IL-33, IL-25, HMGB1, IL-1α) whose expression is upregulated only in lesional AD [12] have been utilized in several studies. In skin, alarmins contribute to the recruitment and activation of immune cells. They are produced by KCs after UV irradiation, microbial infections, and wounding via mechanisms that are only partially elucidated. *TSLP* expression has been shown to be enhanced by UV-irradiation in a dose-dependent manner in HEEs via the stabilization of hypoxia-inducible factor (HIF)-1α by phosphorylated extracellular signal-regulated kinases (ERK) and c-Jun N-terminal kinases (JNK) [106]. Moreover, exogenous IL-1β (60 ng/mL) in combination with TLR2/3 ligands (5–10 μg/mL), stimulated the production of TSLP at the mRNA and protein levels in HEEs generated with KCs isolated from healthy and AD skin [107]. This was, at least in part, mediated via the activation of the NF-κB pathway [107]. These studies show that augmented TSLP in lesional AD skin might not only result from impaired epidermal barrier per se, but also from subsequently increased sensitivity to UV via distinct signaling pathways.

Addition of IL-33 to HEEs had very targeted effects as it specifically downregulated *LCE2A*, a marker of KC late differentiation, but had no effect on other epidermal differentiation and inflammation markers in HEEs [104]. In contrast, HMGB1 had broader effects on epidermal differentiation by downregulating FLG, IVL, LOR, and *KLK5*, but had no effect on inflammation [93]. These effects were similar in HSEs, although not identical [93]. Moreover, IL-33 and HMGB1, when added to HEEs, increased KC proliferation, suggesting a role for these inflammatory mediators in epidermal hyperplasia in lesional AD [104]. Thus, these results show that alarmins affect the proliferation/differentiation process in lesional AD, potentially in an effort to restore the epidermis, but do not significantly trigger the release of other inflammatory mediators by KCs. HEEs treated with IL-4, IL-13, and an alarmin, namely IL-25, recapitulated epidermal features of lesional AD such as a widening of the intercellular spaces (spongiosis) and dysregulated expression of AD biomarkers, including a decrease in FLG, LOR, and E-cadherin at the protein and/or mRNA levels, along with an increase in carbonic anhydrase 2, neural epidermal growth factor L-Like protein 2 and hyaluronic acid synthase 3 [105]. These results also corroborate those from a previous study showing an imbalance of hyaluronan synthases 1 and 3 in AD lesions compared with healthy and non-lesional AD skin [108].

### 3.2. JAK Inhibitors Restore FLG and Dampen Th2 Mediators in 3D AD Cultures

Janus kinase (JAK) inhibitors are used to treat cancer and autoimmune and inflammatory diseases. In dermatology, JAK inhibitors seem to be effective in treating alopecia areata [109] and are promising therapeutic options in severe forms of AD and psoriasis. PF-04965842 is a JAK1 inhibitor currently in phase II clinical trials for moderate to severe AD. The relevance of the therapeutic approach using JAK inhibitors is also fueled by several pre-clinical results generated with 3D organotypic cultures. For example, Tofacitinib, a JAK1/3 inhibitor, restored FLG in HSEs treated with IL-4 and IL-13 [103]. Moreover, it suppressed the expression of inflammatory molecules, including *CXCL10*, *IL13R12*, *SOX2*, *IL24*, *IL4R,* and *POSTN*; the last encodes periostin, a protein upregulated in response to Th2 cytokines and which further promotes a Th2 immune deviation (amplification loop) [103,110].

### 3.3. Elucidation of the Role of Microtubules and the F-Actin Cytoskeleton in AD by Using 3D Cultures

Hsu et al. recently proposed a link between AD and microtubules because of the potential involvement of microtubules in the formation of the epidermal barrier [111]. In HEEs treated with pro-inflammatory cytokines mimicking the pro-inflammatory milieu observed in lesional AD skin (i.e., IL-4, IL-13, IL-31, and TNF-α), the amount of acetylated α-tubulin, a marker of stable microtubules, was reduced when compared to cytokine-free HEEs [111]. Interestingly, the regular spacing of LBs at the interface between the SG and the SC was lost and was associated with reduced secretion and amounts of corneodesmosin, an LB marker [111]. Stabilization of microtubules with paclitaxel or epothilone B reversed the detrimental effects of cytokines on the expression of acetylated α-tubulin and LBs. Moreover, paclitaxel or epothilone B was able to trigger epidermal differentiation by increasing FLG and LOR and to augment cell junctions by increasing E-Cadherin and β-catenin [111]. Moreover, data suggest that treatment with the cytokine cocktail interferes with the assembly of both adherens and tight junctions, whereas additional treatment with paclitaxel or epothilone B supports the assembly of junctional proteins by inducing the stabilization of cortical microtubules. Consistent with this, the amount of Lis1, a microtubule organizing protein, was reduced in the epidermis of AD lesions when compared to nonlesional and healthy epidermis [111]. Thus, this work suggests, for the first time, that microtubules are abnormal in the epidermis of AD skin lesions, where they might sustain a dysfunctional epidermal barrier and, in turn, perpetuate the disease [111]. This is in line with more recent work showing a role for actin-based motor myosin Vb (Myo5b) in maintaining epidermal barrier integrity by controlling LB secretion [53]. Indeed, HEEs silenced for *MYO5B* displayed epidermal barrier defects associated with structural alterations of the SC and a reduced LB pool, resembling observations made in AD [53]. This work strongly suggests that LB distribution in granular KCs might be dependent on a dynamic F-actin cytoskeleton [53].

### 3.4. Histamine Induces Cellular Abnormalities in 3D Cultures Resembling Defects in AD

Mast cells are present in healthy skin but massively infiltrate the dermis of patients with AD. When activated, they release important signaling molecules such as prostaglandin D_2_ and histamine, both having potent pro-inflammatory effects. After mast cell degranulation, histamine concentrations within the skin can rise to 10–1000 μM [112]. Increased histamine levels have been reported in lesional and nonlesional skin of patients with AD but their effects on KCs remained unclear until only recently. Gschwandtner et al. reported that HSEs treated with histamine (100 nM–10 mM) displayed an impaired tight junction barrier (inside-out barrier), as shown by the diffusion of EZ-Link Sulfo-NHS-LC-LC-Biotin to the SC, and reduced amounts of key proteins of tight junctions such as OCLN and CLDNs. This resulted from inhibition of KC differentiation and SC formation [113]. Thus, release of histamine by dermal mast cells in AD skin can not only drive skin itchiness, but also significantly contribute to the perpetuation of cellular abnormalities, hence aggravate the disease.

### 3.5. Coal Tar Alleviates Cellular Abnormalities in AD 3D Cultures

Aryl hydrocarbon receptor (AHR) is a cytoplasmic transcription factor that translocates to the nucleus upon ligand recognition, where it binds to specific motifs in the DNA known as xenobiotic response elements. AHR is crucial in regulating the development of lymphoid cells and the induction of regulatory T cells and is also expressed in the epidermis, especially in the SG [114]. Recently, the environmental pollutant and AHR ligand tetrachloro-dibenzo-dioxin (TCDD) was described to induce epidermal differentiation. Bogaard et al. showed that by treating HSEs with coal tar that contains, inter alia, and AHR ligands, epidermal differentiation was accelerated via the upregulation of FLG and LOR. Moreover, coal tar upregulated FLG in HSEs generated with primary cells isolated from AD patients heterozygous for *FLG* null mutations. Furthermore, the same authors treated HSEs with IL-13 and IL-4 to induce spongiosis in order to mimic lesional AD. Coal tar was able to reduce both spongiosis and the level of *CCL26*, a chemokine involved in eosinophil chemoattraction in AD. The authors hypothesized that AHR ligands contained in coal tar are able to upregulate FLG, HNRN, and IVL and reduce spongiosis and oxidative stress induced by Th2 cytokines via the activation of nuclear factor (erythroid-derived 2)-like (NRF) 2 [115]. Nevertheless, the role of increased Th2 cytokine-mediated oxidative stress in AD remains to be evidenced. Moreover, overexpression of constitutively activated AHR in basal epidermal KCs in mice led to AD-like inflammation [116,117]. Recently, it was shown that *Staphylococcus epidermidis* is able to trigger IL-1β and IL-1α in an AHR-dependent manner in HSEs, suggesting a pro-inflammatory role of AHR [118]. This proves the complexity of the mechanism of action of such transcription factors. It is now well established that both the nature and the dose of ligands and whether they are applied alone or in combination with other compounds can lead to different metabolic responses [119,120]. Coal tar contains approximately 10,000 chemicals, of which only about 50% have been identified. Thus, further studies are required to better understand AHR function in the skin and the conditions under which it exerts beneficial or detrimental effects.

### 3.6. Toward More Complex 3D AD Cultures

Engineering more complex 3D cultures is an important challenge if we want to eliminate the use of animal models to study AD. Such cultures will also help in the collection of important clinically relevant data. Roggenkamp et al. have developed a model of reconstructed skin comprising human dermal fibroblasts and KCs and porcine dorsal root ganglia neurons. These HSEs displayed neurites in the dermal equivalent, as well as beneath the epidermis, resembling skin innervation in vivo. In addition, thin nerve endings ascended into the epidermis, where they showed axonal varicosities in close proximity to KCs [121]. By utilizing skin fibroblasts and KCs from AD patients, the authors showed that innervation was able to induce epidermal hyperplasia—AD cells without innervation did not [121]. Moreover, they showed that innervated AD HSEs exhibited increased neurite outgrowth when compared to HSEs generated with cells from healthy donors. Furthermore, Calcitonin Gene-Related Peptide (CGRP) and Substance P (SP) both enhanced KC proliferation, pointing to a correlation between epidermal hyper-innervation and hyperplasia [121]. CGRP and SP are two neuropeptides initializing neurogenic inflammation, a term coined long ago to describe neurogenic mast cell degranulation with subsequent vascular responses [122]. Today, neurogenic inflammation is seen as a more complex process in which the pro-inflammatory effects of neuropeptides on mast cells, T cells, and KCs supersede their anti-inflammatory or tolerogenic effects on dendritic cells. This is of physiopathologic relevance because elevated levels of CGRP and SP have been found in suction blister fluids from AD patient skin [121]. Thus, this work helped reveal new crosstalk involving the peripheral nervous system, epidermal morphogenesis, and homeostasis, which could have relevance in skin diseases such as AD where stress is a key etiologic factor.

### 3.7. New Pathogenic Pathways Related to Eicosanoids Illuminated with 3D AD Cultures

We have generated HEEs using AD patient KCs with [AD (FLG/WT)] or without [AD (WT/WT)] *FLG* null mutations isolated from nonlesional trunk skin (Figure 1). SC from AD (WT/WT) and AD (FLG/WT) HEEs showed disorganized lamellar bilayer structures, evidenced by incompletely processed lamellar material, similar to the defect observed in IV HEEs [49]. Moreover, there was premature and inhomogeneous secretion of LB contents into the extracellular space. LB entombment, i.e., entrapment of nonsecreted LB contents within the corneocytes, was seen in approximately 50% of AD HEEs. Together, these ultrastructural analyses confirmed that HEEs generated with AD KCs recapitulated the structural abnormalities reported in AD human skin ([123] and unpublished data). Although LBs were abnormal in AD HEEs, the permeability barrier to LY and testosterone was not altered, demonstrating no major impairment of the SC barrier [49]. Moreover, we showed that HEEs generated with primary AD KCs conserved other essential AD hallmarks such as enhanced expression of pro-inflammatory cytokines, including *IL1B*, *TNFA,* and *CCL17*. In contrast, *IL33* and *TSLP* mRNA levels were not upregulated [49]. Because these HEEs faithfully recapitulated AD features, we used them to investigate unexplored pathways that are altered in AD. We showed an imbalance in the metabolic synthesis of ω6-polyunsaturated fatty acids (PUFAs) in AD (FLG/WT) HEEs, which could be explained by a potential increase in the expression of several PLA_2_ isoforms (*PLA2G2A*, *PLA2G2F,* and *PLA2G3*). Among the ω3-PUFAs, the levels of docosapentaenoic acid (DPA), eicosapentaenoic acid (EPA), and docosahexaenoic acid (DHA) were higher in AD (WT/WT) and AD (FLG/WT) HEEs when compared with control HEEs. Moreover, we showed that arachidonic acid (AA) and 12-HETE accumulated in AD (FLG/WT) HEEs when compared to AD (WT/WT) and control HEEs. Further experimentation showed that AA promoted the expression of *IL1B* and *CCL17*, whereas AA-derived 12-HETE dampened the expression of *HRNR* in HEEs [49]. Hence, the accumulation of AA and 12-HETE in AD skin might synergize to trigger both inflammation and SC abnormalities [49]. Furthermore, this work emphasizes the power of HEEs generated with primary patient KCs to unravel new pathogenic pathways involved in AD.

**Figure 2:**
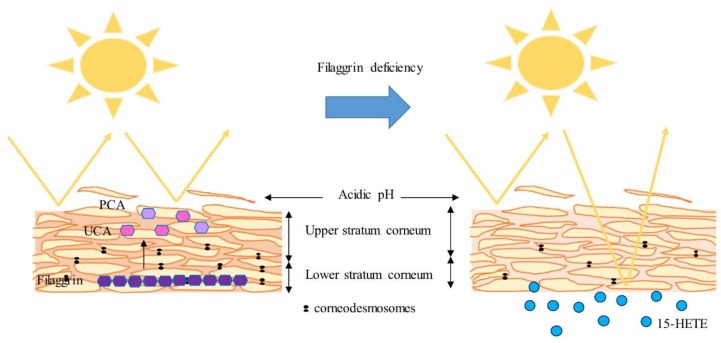
Lessons from IV 3D cultures. *trans*-urocanic acid (UCA) and pyrrolidone carboxylic acid (PCA) produced by Filaggrin (FLG) proteolysis likely serve as natural sunscreens. Accumulation of 15-HETE in IV epidermis might limit the extent of damage inflicted by UV-irradiation. FLG does not significantly contribute to the acidic pH of skin. FLG deficiency leads to an abnormal, but not completely defective, stratum corneum (SC) and has little or no effect on the keratinocyte (KC) differentiation process.

In summary, recent work carried out with organotypic culture models has proven their value by unraveling new cellular and molecular abnormalities involved in ichthyoses and AD, rejecting several hypotheses and testing new therapeutic approaches. Indeed, HEEs and HSEs have helped to underscore the role of FLG as a substrate for the synthesis of components of NMFs, namely UCA and PCA, which likely serve as natural sunscreens. Of note, considerable focus has been placed on understanding the role of FLG in AD, even sometimes incautiously by extrapolating results obtained in FLG knocked down models to AD, when they are in fact models for IV. Nevertheless, FLG deficiency would thus lead to increased vulnerability of the epidermis to UV irradiation by loss of UCA and PCA. However, accumulation of 15-HETE in IV epidermis might limit the extent of damage (Figure 2). Recent work has not supported a role for FLG in contributing significantly to the acidic pH of skin, as was initially postulated. Moreover, conclusions drawn from these models clearly established that FLG deficiency leads to an abnormal, but not defective, SC and has little or no effect on the KC differentiation process. In contrast, the use of Th2 cytokine and alarmin cocktails in HEEs and HSEs demonstrated that the specific pro-inflammatory milieu found in AD skin leads to dysregulation of KC proliferation and differentiation and to alkalinization of the epidermis. Furthermore, the accumulation of specific lipids, namely 12-HETE and AA, in AD epidermis might significantly contribute to disease pathogenesis or perpetuation by, respectively, dampening KC differentiation and promoting the secretion of pro-inflammatory mediators by KCs, thus emphasizing potential synergetic effects of these two eicosanoids. In addition, new groundbreaking work suggests a role of microtubules and F-actin filaments in the pathogenesis of AD. All this research should stimulate the discovery of new therapeutic targets to treat both IV and AD.

In conclusion, human organotypic skin and epidermis models advantageously recapitulate key features of IV and AD skin, irrespective of the way they are constructed (gene knockdown, addition of cytokine cocktails, primary patient cells). Thus, they are very attractive tools to study these diseases and the efficacy of treatments in pre-clinical trials. However, the efficacy of gene silencing and the quantity of pro-inflammatory cytokines need to be further refined to better reflect clinical observations. Moreover, engineering the models to include several skin cell types (e.g., immune and sensory cells), albeit a great challenge, will help propel new discoveries that may lead to efficacious treatments for ichthyoses and AD.

## Abbreviations

AA	arachidonic acid
AD	atopic dermatitis
AHR	aryl hydrocarbon receptor
AMP	antimicrobial peptide
ARCI	autosomal recessive congenital ichthyosis
CE	cornified envelope
CER(EOS)	ester-linked omega-hydroxy sphingosine
CER(EOdS)	ester-linked omega-hydroxy dihydrosphingosine
CGRP	calcitonin gene-related peptide
CLDN	claudin
DHA	docosahexaenoic acid
DPA	docosapentaenoic acid
EGF	epidermal growth factor
EPA	eicosapentaenoic acid
ERK	extracellular signal-regulated kinases
FFA	free fatty acid
FLG	filaggrin
HBD	human β-defensin
HEE	human epidermal equivalent
HMGB1	high mobility group protein B1
HPETE	hydroperoxyeicosatetraenoic acid
HRNR	hornerin
HSE	human skin equivalent
HIF-1α	hypoxia-inducible factor-1α
iPSC	pluripotent stem cell
IV	ichthyosis vulgaris
IVL	involucrin
IL	interleukin
JAK	janus kinase
JNK	c-Jun N-terminal kinases
KC	keratinocyte
KRT	keratin
LB	lamellar body
LCE	late cornified envelope
LOR	loricrin
LOX	lipoxygenase
LY	lucifer yellow
Myo5b	actin-based motor myosin Vb
NF-κB	nuclear factor ’kappa-light-chain-enhancer’ of activated B-cells
NHE-1	sodium-hydrogen antiporter 1
NMF	natural moisturizing factor
NRF2	nuclear factor (erythroid-derived 2)-like 2
OCLN	occludin
PCA	pyrrolidone carboxylic acid
PUFA	polyunsaturated fatty acid
SC	stratum corneum
SG	stratum granulosum
shRNA	short hairpin RNA
siRNA	small interfering RNA
SP	substance P
sPLA_2_	secretory phospholipase A_2_
TCDD	tetrachloro-dibenzo-dioxin
TEER	transepithelial electrical resistance
TEWL	transepidermal water loss
TGM	transglutaminase
TLR	toll-like receptor
TSLP	thymic stromal lymphopoietin
UCA	trans-urocanic acid
UV	ultraviolet
ZO-1	zonula occludens-1
